# Cloning, expression and location of RNase9 in human epididymis

**DOI:** 10.1186/1756-0500-1-111

**Published:** 2008-11-10

**Authors:** J Liu, JY Li, HY Wang, CL Zhang, N Li, YQ Lin, J Liu, WT Wang

**Affiliations:** 1 Shandong Research Center of Stem Cell Engineering, Yantai Yuhuangding Hospital, Yantai, PR China

## Abstract

**Background:**

Mammalian spermatozoa become fully motile and fertile during transit through the luminal fluid of the epididymis. At least 200 proteins are present in the epididymal lumen, but the potential roles of these luminal proteins in male fertility are unknown. Investigation of the function of these proteins will elucidate the mechanism of sperm maturation, and also provide new drug targets for male contraception. We cloned RNase9 from a human epididymis cDNA library for characterization and analysis of its functions.

**Findings:**

It was predicted that human *RNase9 *gene was located on chromosome 14q11.2 and encoded a 205 amino acids protein with a signal peptide of 26 amino acids at the N-terminus. The protein had eight conserved cysteine residues characteristic of the RNase A family members and several potential post-translational modification sites.

At the transcriptional level, *RNase9 *was expressed in a wide variety of tissues, and the expression was higher in men than in boys. *RNase9 *was localized to the post-equatorial region of the sperms' head. Immunofluorescence staining showed that RNase9 protein was present mostly in the epithelium of the epididymal tubule. Recombinant RNase9 had no ribonuclease activity. In addition, RNase9 had no detectable effect on sperm motility and fertilization as demonstrated by blocking spermatozoa with anti-RNase9 polyclonal serum.

**Conclusion:**

*RNase9 *is expressed in a wide variety of tissues. It is located on the post-equatorial region of the sperm head and the epithelium of epididymal tubule. Although *RNase9 *belongs to the RNase A family, it has no ribonuclease activity.

## Background

The epididymis is a long, convoluted duct. As immature spermatozoa move through the epididymis, they are transformed into fully motile and fertile sperm cells through sequential interactions with proteins present in the luminal fluid of the epididymis [1]. At least 200 proteins are present in the epididymal lumen [2]; however, the *in vivo *function of most of these proteins is unclear. Exploring the function of these epididymal proteins will be necessary for a full understanding of sperm maturation and potentially will lead to the identification of new drug targets for male contraception. *RNase9 *initially was identified in mouse epididymis through *in silico *analysis of expressed sequence tags (ESTs) in the UniGene library collection. Later, Devor et. al. identified human *RNase9 *in the human genome assembly, but the function of this gene is unknown and the characterization has yet to be done.

## Methods

### Sequence analysis, cloning and expression of rRNase9 protein

GENSCAN , Genome Browser , SignalP3.0 , Profile-Scan  and MacVector software were used in this study. The cDNA fragment encoding the mature RNase9 peptide was amplified from a human epididymis cDNA library that we constructed [3]. With the following gene specific primers: forward primer F 5'-*TTGGTAC *CGA CGA CGA CGA CTG GTG CAG TTT CAA GAG GTG-3' (adds a KpnI site on its 5'end) and the reverse primer R 5'-*CCGGAATTC *TCC CCC GGG CTA GGG CGA TAT GA-3' (adds an *EcoRI *site at the 3'end of the amplicon). The fragment was sequenced and inserted into the pET-32b (+) vector that encodes a His epitope tag (Novagen, Germany). Transformed *E. coli *were grown to mid-log phase and fusion protein expression was induced with 1 mM isopropyl-1-thio-β-D-galactoside (IPTG) for 3 h at 37°C. Fractions were analyzed on 15% SDS-polyacrylamide gels (SDS-PAGE), and the molecular weight of RNase9 protein was identified by MALDI-TOF. The recombinant protein was purified by Ni^2+^-affinity chromatography.

### The tissue expression profiles of RNase9

Tissue samples (epididymis, heart, lung, liver, spleen, kidney, stomach, testis, and muscle) were obtained from individuals at different ages (fetus, adult, geriatric). All samples were approved by the Ethics Committee of Affiliated Yu-Huang-Ding Hospital of Qingdao University. Total RNA was extracted with Trizol reagent (Invitrogen, Carlsbad, CA) and 1 μg RNA was reverse transcribed using 20 U AMV Reverse Transcriptase (Promega, Beijing, China) and 0.3 μg of oligo d_T_18 (Promega, Beijing, China) according to the manufacturer's instructions. The human *RNase9 *cDNA fragment was amplified with forward primer F 5'-CTG GTG CAG TTT CAA GAG GTG-3'; reverse primer R 5'-TCC CCC GGG CTA GGG CGA TAT GA-3'. β-actin served as an endogenous control and the primers sequences were as follows: forward primer F 5'-CCA TGC CAA TCT CAT CTT-3'; reverse primer R 5'-CGT GAC ATT AAG GAG AAG-3'. The PCR reaction was carried out with Ex-Taq (TAKARA, Japan) under the following conditions: 30 s at 95°C, 40 s at 56°C (*RNase9*)/49°C (β-actin), and 1 min at 72°C.

### Immunolocalization of RNase9 in spermatozoa

Human semen samples were collected after three days of sexual abstinence from normal fertile donors at 30 years of age. Testicular sperm was isolated using 52% isotonic Percoll (Amersham Bioscience) and resuspended in PBS [4]. Epididymal sperms were recovered from the corpus and caudal regions of the epididymis by puncture of the ductuli and the sperm drops were flushed with PBS containing 1 mM phenylmethylsulfonylfluoride (PMSF). Sperm smears then were blocked with 3% BSA in PBS (PBS-BSA, w/v) for 60 min at room temperature and subsequently incubated overnight at 4°C with human RNase9 antiserum (1:400). Parallel smears incubated with mouse non-immune serum served as a negative control. After three washes with PBS, smears were incubated for 60 min at room temperature with fluorescein isothiocyanate (FITC)-conjugated goat anti-mouse IgG (1:200). Samples were washed in PBS, counterstained with propidium iodide (PI, Invitrogen), and then imaged with a laser scanning confocal microscope (Carl Zeiss, META 510).

### Immunolocalization of *RNase9 *in epididymis and testis

Frozen epididymis and testis were cut into serial 8 μm-thick sections on a Reichert-Jung cryomicrotome and mounted on poly L-lysine-coated slides. The sections were freeze-dried and fixed with 4% paraformaldehyde for 20 minutes. The samples then were permeabilized for 30 minutes with 0.5% Triton X-100 in PBS. Subsequently, tissue sections were blocked for 60 minutes with 3% BSA (w/v) in PBS at room temperature and incubated overnight at 4°C with polyclonal anti-RNase9 serum (1:400). After several washes with PBS, the sections were incubated for 60 minutes at room temperature with FITC-conjugated goat anti-mouse IgG (1:200). As a negative control, serial sections were processed with the same procedure but pre-immune serum was used as primary. After three washes with deionized water, the sections were mounted and examined with a confocal microscope (Carl Zeiss, META 510).

### Ribonuclease activity assay

RNase9 (20 nmol) was incubated with 1.42 nmol of a standard yeast tRNA substrate in 40 mM sodium phosphate buffer (pH 7.4) at 25°C. The reaction was terminated by the addition of 0.5 ml of 20 mM lanthanum nitrate with 3% perchloric acid, and the insoluble tRNA was removed by centrifugation. The amount of solubilized tRNA was determined by ultraviolet absorbance at 260 nm. Catalytic activity was expressed as pmol of RNA digested in 1 sec by 1 pmol of RNase9. Recombinant protein HEL-75 (with no RNase activity) was used to control for bacterial RNase contamination [5]. Three independent experiments were conducted.

### Sperm motility assay and zona-free hamster egg penetration test

Spermatozoa were incubated with RNase9 antiserum (1:200) at 37°C in 5% CO2 for two hrs and then evaluated for motility using the computer-aided sperm analysis (CASA) system. Sperm penetration was determined by the presence of a swollen sperm head within the cytoplasm of a zona pellucida-free hamster egg. The penetration index was calculated by dividing the number of eggs penetrated by the number inseminated [6]. For two groups of data, two-tail Student's tests were used. A probability of P < 0.05 was ceonsidered to be statistically significant.

## Results

**Figure 1:**
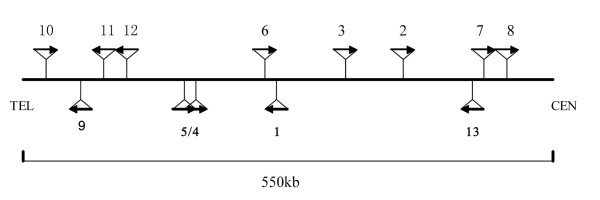
**Diagram of human chromosome 14q11.2.** A 550-kb region of chromosome 14q11.2 containing all 13 members of the RNase A gene family is shown. The relative spacing of *RNase9 *within the RNase A family, as well as their transcription orientations also is shown.

**Figure 2:**
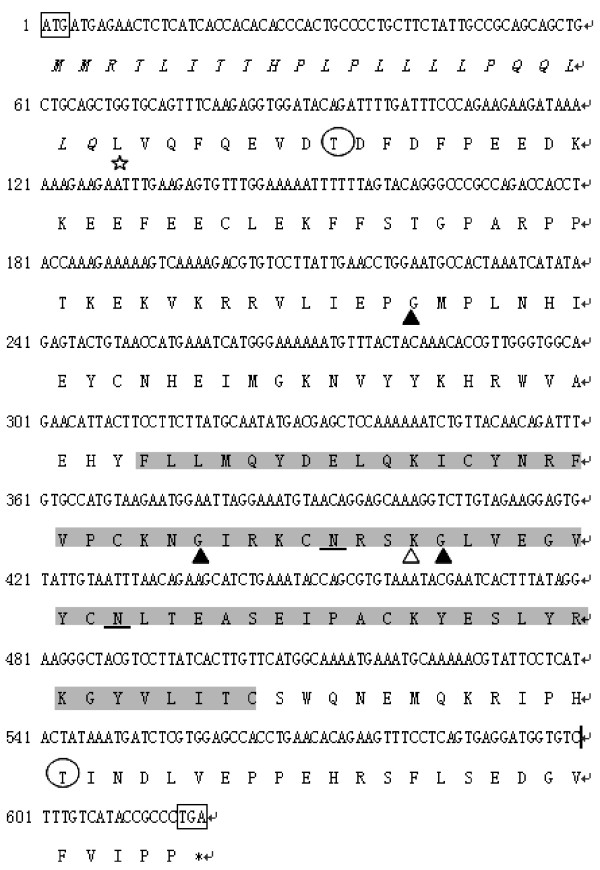
**The cDNA and amino acid sequences of human *RNase9*.** The open reading frame contains 618 bp and encodes a 205-amino acid protein. The initial and terminal codons are boxed. The protein contains a putative signal peptide with a cleavage site between L_23 _(star) and V_24_. Predicted N-glycosylation sites (at N_131 _and N_143_; underscored), casein kinase II phosphorylation sites, (T_31_DFD and T_181_IND; circled), N-myristoylation sites (G_74_MPLNH, G_126_IRKCN, and G_135_LVEGV; solid triangles), and a tyrosine kinase phosphorylation site (KGLVEGVY_141_, open triangles) are shown. The location of the putative Big-1 domain (L_23_VQFQ; star) and the pancreatic ribonuclease (shaded) also are shown.

The *RNase9 *gene is located on human chromosome 14q11.2 [GenBank: NT-026437], along with the twelve known members of the RNase A family (Figure 1). *RNase9 *was predicted to encode a protein of 205 amino acids. The first 26 amino acids of RNase9 were identified a putative signal peptide by SignalP3.0 software. After cleavage of the signal peptide, the mature RNase9 protein was 179 amino acids in length, with a pI of 5.92. At the amino acid level, *RNase9 *was 15% ~ 30% identical to the canonical RNase A members *(RNase *1 – 8). Functional sites on the protein were predicted using the software, Profile-Scan . Two potential N-glycosylation sites were predicted at N_131_RSK and N_143_LTE; three N-myristoylated sites were predicted at G_74_MPLNH, G_126_IRKCN, and G_135_LVEGV. In addition, two potential post-translational phosphorylation modification sites were present in this sequence, including two casein kinase II phosphorylation sites, T_31_DFD and T_181_IND, and one tyrosine kinase phosphorylation site, Y_141_. The sequence also contains a potential Big-1 (bacterial Ig-like) domain at L_23_VQFQ (Figure 2).

**Figure 3:**
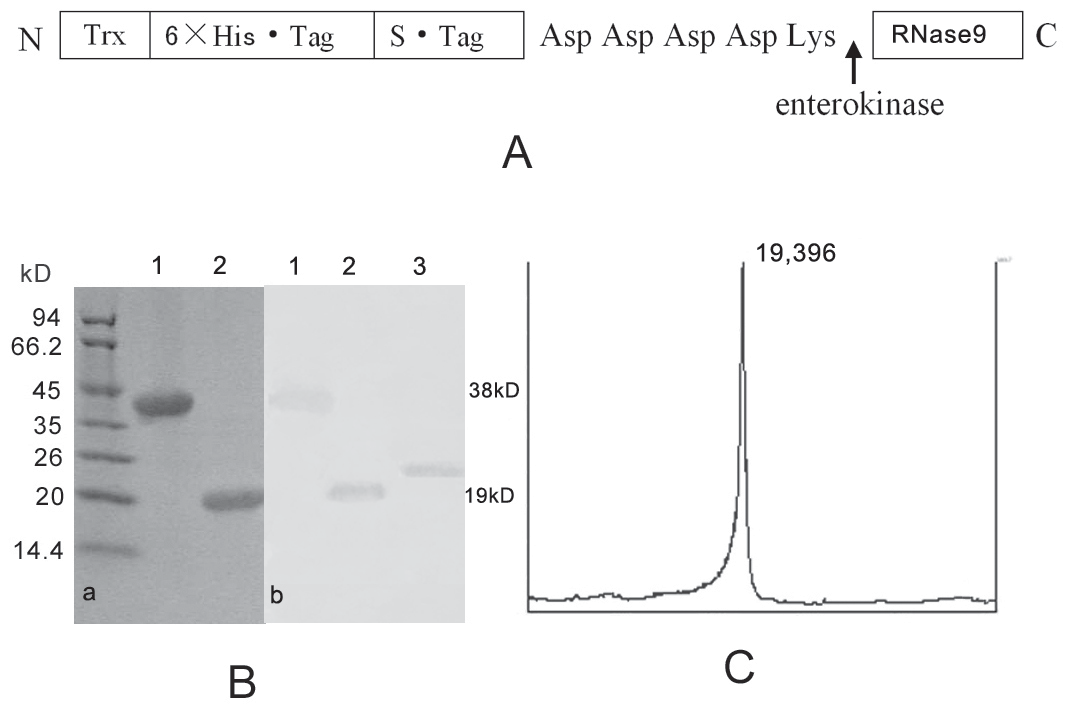
**Identification of rRNase9 and western blot results.** A: Strategy for expressing RNase9 protein as a Trx fusion in *E. coli*; B: (a) SDS-PAGE. 1. Trx-rRNase9; 2. rRNase9; (b)Western blot 1.Trx-rRNase9; 2. rRNase9; 3. RNase9 in epididymal fluid. C: MALDI-TOF analysis of rRNase9.

**Figure 4:**
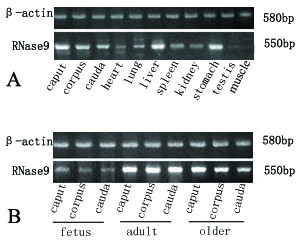
**Expression of *RNase9 *(A) Tissue distribution of *RNase9*.** The indicated tissues were analyzed for *RNase9 *mRNA levels (550 bp) by RT-PCR. RNA (2 ug) from various tissues and different parts of the human epididymis was reverse-transcribed and analyzed by RT-PCR.*RNase9 *was expressed in all the tissues: epididymis, heart, lung, liver, spleen, kidney, stomach, and testis except muscle (negative control). *β-actin *(580 bp) served as an internal control. (B) *RNase9 *expression in epididymis. RT-PCR analysis of *RNase9 *mRNA levels (550 bp) in human epididymal tissues. Human epididymis tissues were collected from fetus, adult, and older individuals. *β-actin *(580 bp) served as an internal control.

**Figure 5:**
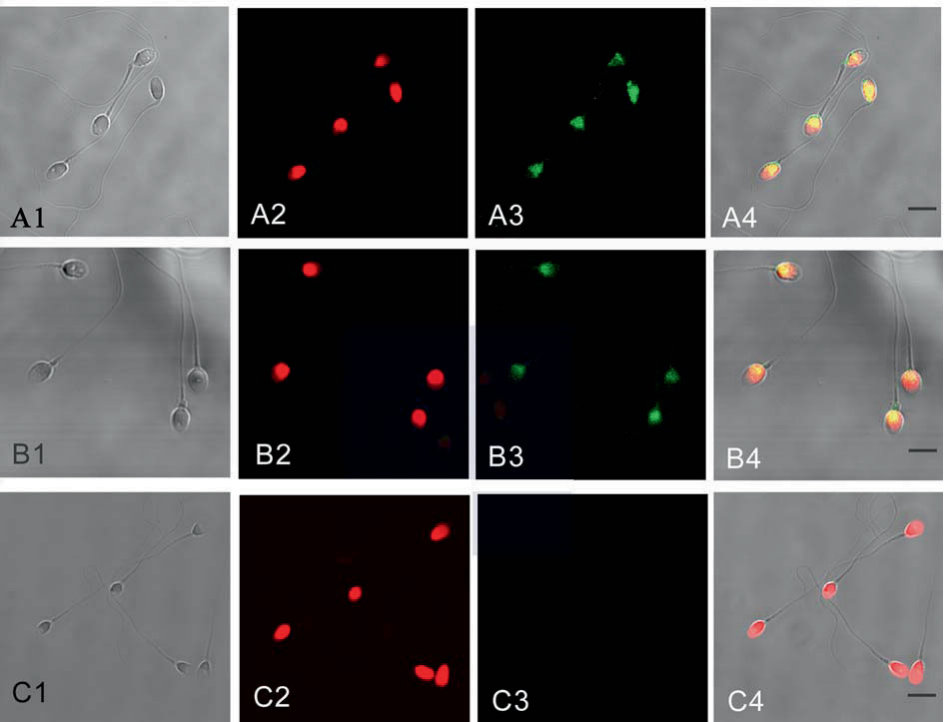
I**mmunofluorescence staining of RNase9 in spermatozoa.** A1~C1: phase contrast image; A2~C2 nuclear counterstain (PI); A3: Ejaculated sperm; B3: Epididymal sperm; C3: Testicular sperm; A4~C4: Merged image; Green staining indicates the immunofluorescence of human RNase9 (FITC-labeled); Red staining (PI) indicates the nuclei. Scale bar: 5 μm.

**Figure 6:**
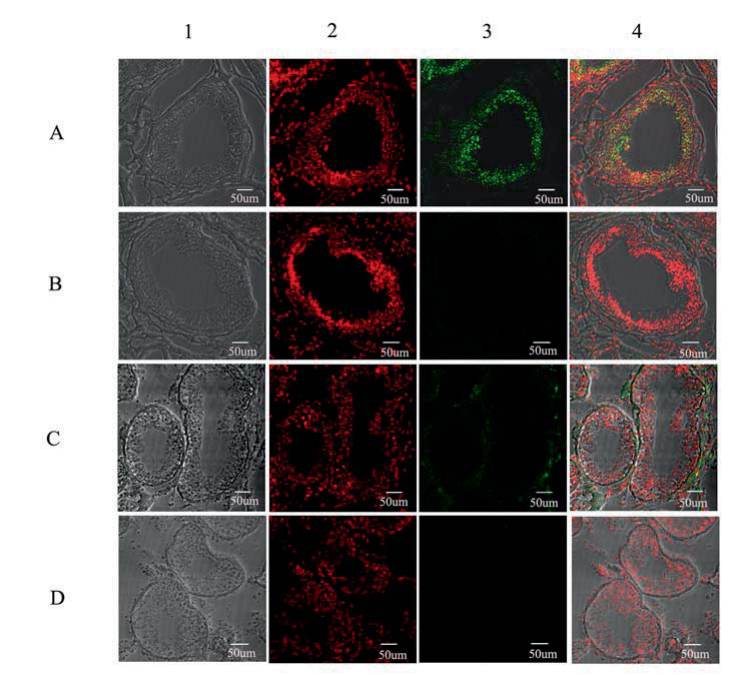
**Immunofluorescence staining of RNase9 in epididymis and testis. **A, B: epididymis tissue; C, D: testis tissue; B, D: negative control. 1: phase contrast image; 2: nuclear counterstain (PI); 3: Green immunofluorescence staining of human RNase9 (FITC-labeled); 4: Merged image. Scale bar: 50 μm

**Figure 7:**
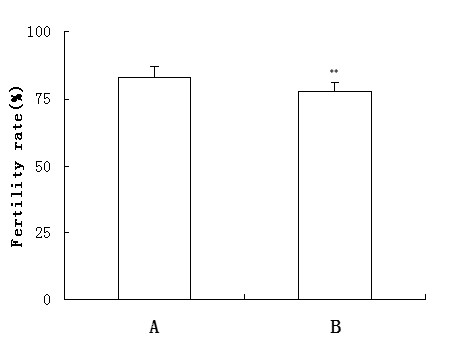
**Zona-free hamster egg penetration test.** In vitro fertilization test, the total number of eggs tested was 60(A group) and 62 (B group), respectively. Fertility rate has no significant difference between A (50 of 60, 83%) and B (48 of 62, 77%) group, **p > 0.05. A: Sperm blocked with pre-immune serum (1:200) for 5 h as control; B: Sperm blocked with RNase9 anti-serum (1:200) for 5 h.

We prepared the recombinant RNase9 protein through a fusion expression strategy (Fig. 3A). The fusion tag can be released easily with the enterokinase site fused between the tag and target protein. SDS-PAGE and MALDI-TOF showed the mass of RNase9 protein was 19 kD, consistent with the theory (Fig. 3B, C). Western blot analysis also indicated that the natural RNase9 protein was slightly larger than the recombinant RNase9. *RNase9 *was expressed in all tissues (epididymis, heart, lung, liver, spleen, kidney, stomach, and testis) except muscle (Figure 4A). Furthermore, expression of RNase9 was higher in adults than in elderly men or boys (Figure 4B). Indirect immunofluorescence staining showed that RNase9 protein was localized predominantly to the post-equatorial region of the sperms' head: approximately 80% of the epididymal and ejaculated spermatozoa were positive (Figure 5). RNase9 protein signal was detected in the epithelial cells of the epididymal tubule and no fluorescence was detected in the testis (Figure 6). RNase9 protein had no detectable RNase activity towards yeast tRNA (Table 1). The enzyme activity of RNase 1 was determined under the same experimental conditions with the same amount of substrates in order to obtain a direct comparison. Meanwhile, we used the recombinant HEL-75 protein as a control to eliminate the possibility of contamination by bacterial RNase. Comparing the two group spermatozoa that were blocked with RNase9 anti-serum and pre-immune serum respectively, there was no significant difference in motility and fertility of sperm (p > 0.05) (Table 2, Figure 7).

**Table 1:** Ribonuclease activity

Enzyme	Activities* ± SD
RNase1	0.167 ± 0.002^†^
RNase9	0.011 ± 0.007^†^
HEL-75	0.002 ± 0.001^†^

*Ribonuclease activities were measured as pmol RNA digested per pmol enzyme per second. ^†^Value represents the mean of three independent experiments.

**Table 2:** Motility parameters analysis of sperm

Parameters	A	B
VAP(um s-1)	33.80 ± 0.97	36.31 ± 1.52*
VSL(um s-1)	33.34 ± 1.11	30.82 ± 1.89*
VCL(um s-1)	48.19 ± 1.07	45.75 ± 0.86*

N = 10. Data were obtained by using computer assisted sperm analysis and are expressed as mean ± SEM. *p > 0.05. A: Sperm blocked with pre-immune serum (1:200) for 5 h as control; B: Sperm blocked with RNase9 anti-serum(1:200) for 5 h. VAP, avaraged path velocity; VSL, straight line velocity; VCL, curvilinear velocity.

## Discussion

A large number of genes have been identified with the advance of technology, but human epididymis studies are constrained by the impracticality of experimentation and by the advanced age of available tissue donors. To overcome these difficulties, we constructed a normal adult human epididymis cDNA library[3] and cloned *RNase9*. Human *RNase9 *share homology with mice, rats, monkeys, and chimpanzees and belongs to the RNase A family. However, *RNase9 *lacks the conserved catalytic triad and the family's signature motif (CKXXNTF) [7]. This may explain the loss of ribonucleic activity with purified RNase9 protein. In addition, RNase9 was found to have a pI that is lower than that of other RNase A family members. The relatively high pI of typical RNases is thought to be important for their ribonucleic activity, since the positive charge increases the affinity of the enzyme to the negatively charged RNA substrate [8].

Previous studies showed that most RNase genes are expressed in a wide range of tissues except for RNase 3 and RNase 8, which are expressed prominently in eosinophils and placenta, respectively [9,10]. Likewise, human *RNase9 *also is expressed in many tissues. In contrast, rat *RNase9 *is located on chromosome 15 and expression was localized to the epididymis. There are no other reports about RNase9 at present.

In the sperm, the postacrosomal region or equatorial segment is involved in sperm-egg plasma membrane fusion. Indirect immunofluorescence demonstrated that the RNase9 protein is expressed on the post-equatorial region of the sperm head. Taken together with our RT-PCR results and immunofluorescence staining results, these findings suggest that the RNase9 protein is synthesized maily by the epididymis and always binds to the surfaces of epididymal and ejaculated spermatozoa. We know that the sperm membrane undergoes substantial remodeling to induce sperm maturation during epididymal transit and many epididymal proteins take part in this process. Further study into the actual roles of RNase9 protein is needed.

Many proteins in the epididymis are androgen-regulated, such as β-defensin 118 (DEFB118), cystatin-related epididymal specific (CRES) protein, disintegrin and metalloprotease (ADAMs) [11,12]. Rat *RNase*9 is also androgen dependent. At present, the relationship between human RNase9 and androgens is unknown and the castration test is limited by experimental conditions, but RT-PCR results in different individuals showed indirectly that the expression of *RNase9 *was highest in adults. Whether the expression of *RNase9 *is androgen-regulated is not known and requires further study. Additional approaches are under development to analyze the reproductive biology in *RNase9 *knockout animal models.

## Authors' contributions

J L was responsible for gene cloning, recombinant protein expression, antibody preparation, ribonuclease activity assay and wrote the manuscript. JYL, HYW supervised the work, participated in experimental design, and helped in manuscript preparation. CLZ conducted the recombinant protein purification. YQL, NL, JL and SHJ contributed to RNA extraction, RT-PCR, immunofluorescence staining. WTW performed the sperm motility and zona-free hamster egg penetration test. All authors have read and approved the final manuscript.
